# Time-Series Analyses of Transcriptomes and Proteomes Reveal Molecular Networks Underlying Oil Accumulation in Canola

**DOI:** 10.3389/fpls.2016.02007

**Published:** 2017-01-10

**Authors:** Huafang Wan, Yixin Cui, Yijuan Ding, Jiaqin Mei, Hongli Dong, Wenxin Zhang, Shiqi Wu, Ying Liang, Chunyu Zhang, Jiana Li, Qing Xiong, Wei Qian

**Affiliations:** ^1^College of Agronomy and Biotechnology, Southwest UniversityChongqing, China; ^2^Engineering Research Center of South Upland Agriculture of Ministry of EducationChongqing, China; ^3^College of Plant Science and Technology, Huazhong Agricultural UniversityWuhan, China; ^4^Department of Computer Science and Technology, Southwest UniversityChongqing, China

**Keywords:** *Brassica napus*, molecular network, lipid metabolism, transcriptome, proteome, seed development

## Abstract

Understanding the regulation of lipid metabolism is vital for genetic engineering of canola (*Brassica napus* L.) to increase oil yield or modify oil composition. We conducted time-series analyses of transcriptomes and proteomes to uncover the molecular networks associated with oil accumulation and dynamic changes in these networks in canola. The expression levels of genes and proteins were measured at 2, 4, 6, and 8 weeks after pollination (WAP). Our results show that the biosynthesis of fatty acids is a dominant cellular process from 2 to 6 WAP, while the degradation mainly happens after 6 WAP. We found that genes in almost every node of fatty acid synthesis pathway were significantly up-regulated during oil accumulation. Moreover, significant expression changes of two genes, acetyl-CoA carboxylase and acyl-ACP desaturase, were detected on both transcriptomic and proteomic levels. We confirmed the temporal expression patterns revealed by the transcriptomic analyses using quantitative real-time PCR experiments. The gene set association analysis show that the biosynthesis of fatty acids and unsaturated fatty acids are the most significant biological processes from 2-4 WAP and 4-6 WAP, respectively, which is consistent with the results of time-series analyses. These results not only provide insight into the mechanisms underlying lipid metabolism, but also reveal novel candidate genes that are worth further investigation for their values in the genetic engineering of canola.

## Introduction

Canola (*Brassica napus L.*), characterized by low erucic acid (<2%), was developed through conventional plant breeding from *B. napus* (Niu et al., 2009). Canola is one of the most important oilseed crops in the world, and canola oil has been considered healthier than natural rapeseed oil. According to USDA (United States Department of Agriculture), rapeseed/canola oil is now the third largest vegetable oil by volume after palm and soybean oil^1^. With the ever-increasing demands for oil, numerous attempts have been made to improve the yield and/or modify the composition of oil by engineering oil crops genetically (Fujisawa et al., 2009; Voytas and Gao, 2014). However, the inadequate knowledge on the regulation of lipid metabolism severely hinders the process (Bates and Browse, 2011). Therefore, the genetic improvement may be greatly accelerated from the comprehensive understanding to the regulation mechanisms underlying the lipid metabolism at the network level.

Lipid metabolism is one of core biological processes in developing oilseeds. High-throughput gene expression profiling technologies, especially RNA sequencing, have been increasingly utilized to identify the key genes involved in lipid metabolism or seed development for various plants, such as soybean (Chen et al., 2012; Goettel et al., 2014), peanut (Yin et al., 2013), castor (Brown et al., 2012; Zhang et al., 2016), maize (Lu et al., 2013), *Arabidopsis* (Le et al., 2010; Belmonte et al., 2013), and rapeseed (Hu et al., 2009; Troncoso-Ponce et al., 2011; Chen et al., 2015; Deng et al., 2015; Xu et al., 2015). Although, considerable research has been devoted to study the molecular mechanism underlying lipid metabolism in various plants, little effort has been made to investigate, at the whole-genome level, dynamic changes of oil biosynthesis genes during seed development in *B. napus*, which is different from other oil plants in terms of oil content and oil composition. Moreover, in previous studies, there exist inconsistent observations regarding the temporal changes in expression levels of genes related to oil biosynthesis in *B. napus*. Troncoso-Ponce et al. (2011) reported a decline in relative abundance of expressed sequence tags for many oil biosynthesis enzymes during seed development in *B. napus*, while other studies indicated a bell-shaped temporal expression pattern (Hu et al., 2009; Deng et al., 2015). More investigations are required to resolve the dispute.

Transcript abundance on its own might not be enough for inferring the metabolic activity in central plant metabolism due to various factors such as post-transcription machinery and different half lives (Schwender et al., 2014). Proteomic analysis bridges the gap between our understanding of genome sequence and cellular behavior since it detects a complete set of proteins that is expressed. However, proteome-wide analyses of developing seeds of *B. napus* have been rarely reported. A study (Hajduch et al., 2006) has analyzed the developing *B. napus* seeds and identified multiple distinct patterns related to different cellular processes, but it didn’t give a fine-grained view of the proteome profile because only a limited number of proteins were examined. An integrative analysis of transcriptomic and proteomic changes more comprehensively characterizes the dynamic process regulating lipid metabolism than a single data analysis, however, a joint time-series analysis of transcriptomic and proteomic data has not yet been reported for developing seeds in *B. napus*.

In this study, through time-series analyses of transcriptomes and proteomes of canola, we aim to (1) systematically identify the core biological processes involved in oil accumulation and seed development; (2) characterize the dynamic changes in gene networks regulating the biosynthesis and degradation of fatty acids (FA); (3) identify key genes that are potential targets for the genetic improvement of the yield and composition of canola oil. Except for some well-known lipid-related genes, we also identified a large number of novel candidate genes possibly responsible for lipid metabolism, which are worth further investigation for their values in the genetic engineering of oil crops.

## Materials and Methods

### Characterization of Developmental Profiles of Oil Accumulation and Fatty Acids

The developing seeds at 2, 4, 6, and 8 WAP from a canola variety, Zhongshuang 11, with high oil content and high seed yield in the Yangtse River region in China (Wang et al., 2008) were employed for the quantification of developmental profiles of oil accumulation and fatty acids. 2-8 WAP covers the key period of oil accumulation in *B. napus* seed development, which is approximately 2 months in our area. The content of oil and fatty acids were measured using gas chromatography–mass spectrometry (GC–MS) according to Li-Beisson’s method (Li-Beisson et al., 2013). Briefly, 10 mg of sample were heated at 90°C in 1 mL 2.5% (v) H_2_SO_4_ in methanol [containing 20 μg C17:0 (triheptadecanoin, as internal standard)] for 90 min in screw-capped tubes. After the addition of 500 μL of hexane containing 0.01% butylated hydroxytoluene, fatty acids were extracted into the organic phase by shaking and the tubes were centrifuged at low speed. 5 uL of the organic phase were separated by gas chromatography on an Agilent J&W GC column (USA) (30 m by 0.25 mm, 0.25 um film) and quantified using a flame ionization detector (FID). The gas chromatograph was programmed for an initial temperature of 180°C for 1 min followed by an increase of 10°C/min to 220°C; this final temperature was maintained for a further 4 min. Fatty acids are identified and determined by comparison of retention times (and also split patterns) to standards (C17:0). The method gives information on fatty acid methyl esters (FAMEs) content and composition; therefore, percent oil by weight = 100 [(4 total mol FAME/3) + total g FAME]/g tissue, where 4 is the relative molecular weight difference between TAG and three moles of FAME.

### Sample Preparation, RNA Sequencing, Quality Control, Alignment, and Quantification

Canola seeds were harvested at 2, 4, 6, and 8 WAP. After being dissected from the siliques, these seeds were frozen in liquid nitrogen immediately and then stored at -80°C. The total RNA from the four samples were extracted and purified using the RNAprep pure Plant Kit [DP 432, Tiangen Biotech (Beijing) Co., Ltd] according to the manufacturer’s protocol. RNA purity was checked with NanoPhotometer^®^ spectrophotometer (Implen, Westlake Village, CA, USA). The concentration was measured with Qubit^®^ RNA Assay Kit and Qubit^®^ 2.0 Fluorometer (Life Technologies, Foster City, CA, USA). The integrity was assessed using the Agilent 2100 Bioanalyzer and RNA Nano 6000 kit (Agilent Technologies, Santa Clara, CA, USA). Finally, 3 μg RNA per sample was used for the subsequent analysis.

cDNA libraries for samples from the four time points of seed development were constructed using NEBNext^®^ Ultra^TM^ RNA Library Prep Kit for Illumina^®^ (NEB, USA) according to the manufacturer’s protocol. Library quality was assessed using the Agilent 2100 Bioanalyzer. The library preparations were sequenced and 100-bp paired-end reads were generated by an Illumina Hiseq 2000 platform. The clean reads were screened from raw sequencing reads by removing reads containing adapter, ploy-N and low quality reads. The clean reads were aligned to the *B. napus* reference genome, downloaded from Genoscope^2^, using the TopHat program^3^. We set Q to 100 and the other parameters as default. The expression levels of all genes from the *B. napus* reference annotation were quantified using htseq-count 0.6.1p2^4^.

### Time-Series Differential Gene Expression Analysis

Time-series differential expression analysis is used to identify genes with significant temporal expression changes in time-course experiments. RNA-Seq raw counts were normalized by the DESeq normalization (Anders and Huber, 2010). Time-series differential expression analysis was carried out using the maSigPro package (Nueda et al., 2014). The *p*-values were corrected for multiple comparisons by the Benjamini and Hochberg false discovery rate (FDR) procedure. We set *Q* = 0.05 and rsq = 0.7 to get significant genes, and we set *k* = 8 to classify significant genes. GO enrichment analyses were performed using BiNGO (Maere et al., 2005). The significance level was set at 0.0001.

### Gene Set Association Analysis

We performed gene set association analysis for three pairs of samples, 4-2, 6-2, and 8-2 WAP, to identify pathways/gene sets significantly changed during seed development. Gene set association analysis was carried out using GSAASeqSP 2.0 (Xiong et al., 2014). RNA-Seq raw counts were normalized by the DESeq normalization (Anders and Huber, 2010). We chose Signal2 Noise for gene-level differential expression analysis and Weighted_KS for gene set association analysis. We set the FDR cutoff to 0.05, namely gene sets with FDR < 0.05 were considered to be statistically significantly changed between the two time points compared. We set permutation type to gene_set. 121 KEGG pathways and 577 gene ontology (GO) biological processes were extracted from AraPath (Lai et al., 2012). Gene sets with less than 15 genes or more than 500 genes in our RNA-Seq data set were filtered to avoid overly narrow or broad functional categories, and this resulted in 83 KEGG pathways and 201 GO biological processes, respectively.

### Proteomic Analysis

The whole proteome of canola seeds, harvested at 2, 4, 6 and 8 WAP, were extracted using the Plant Total Protein Extraction Kit (PE0230) according to the manufacturer’s protocol. Protein concentration was determined by the Bradford assay (Bradford, 1976), using bovine serum albumin (BSA) as standard. The proteins were reduced, alkylated, and digested using Ding’s method (Belmonte et al., 2013). The enzymatic digests were labeled with TMTsixplex Isobaric Mass Tagging Kit according to the manufacturer’s protocol. Four labels were used in this study. The samples from seeds at 2, 4, 6, and 8 WAP were labeled with 126, 127, 128, and 129, respectively, and then mixed at equal amounts.

The labeled peptides were lyophilized and redissolved in buffer A [98% H_2_O and 2% acetonitrile (ACN), pH 10]. High pH RP-HPLC fractionation was performed on an Agilent 1100 Series HPLC system. Guard column (4.6 mm × 12.5 mm, 5-μ) and analytical column (C18, 5 μm, 2.1 mm × 150 mm) were employed. The mobile phase was composed of A (98% H_2_O and 2% ACN, pH 10) and B (10% H_2_O and 90% ACN, pH 10). The pH was adjusted to 10 with NH_3_H_2_O. The flow rate was 0.3 mL/min. LC gradient was set as follows: 3 min, 2% B; 3.01 min, 6% B; 40 min, 25% B; 50 min, 38% B; 50.01 min, 90% B; 60 min, 90% B; 60.01 min, 2% B; 65 min, 2% B. 4.5-min wide fractions were collected from 5 to 50 min for subsequent LC–MS/MS analysis.

The fractions were lyophilized and redissolved in nano-RPLC buffer A [0.1% formic acid (FA), 2% ACN, 98% water]. The samples were loaded onto a trap column (PepMap100, C18, 3 μm, 75 μm × 20 mm, Thermo Scientific) by buffer A at 2 μL/min for 10 min, followed by separation on an analytical column (PepMap100, C18, 2 μm, 75 μm × 150 mm, Thermo Scientific) using an Easy-nLC 1000 System (Thermo Scientific). A gradient of 5–35% buffer B (0.1% FA, 90% ACN, 10% water) over 70 min at a flow rate of 250 nL/min was used for peptide separation. The separated peptides were analyzed with the data-dependent-acquisition mode on a Thermo Scientific Q Exactive Orbitrap mass spectrometer (MS). Up to 10 of the most abundant precursor ions with charge state ≥ 2 from each initial survey scan were automatically selected for fragmentation by higher energy collision dissociation (HCD) with normalized collision energy (NCE) of 27%. The maximum ion injection times for the survey scan and MS/MS scans were 20 and 60 ms, respectively, and the ion target values (AGC) for both scan modes were set to 1 × 10^6^. Dynamic exclusion was set to 18 s.

All MS/MS spectra were analyzed using Proteome Discoverer^TM^ 1.3 software using the SEQUEST search engine. Data were searched against *B. napus* database with a 1% FDR criteria using Percolator. Quantitative analysis of the TMT experiments was performed using the TMTsix-plex quantification method included in Proteome Discoverer 1.3. Protein identification and quantification results are summarized in Supplementary Table S7. We wrote the ratios of 126/126, 127/126, 128/126, and 129/126 as 2/2, 4/2, 6/2, and 8/2 for better interpretation, and we called these ratios as relative expression values of proteins.

### Quantitative Real-Time PCR Analysis

Total RNA was isolated using RNAprep pure Plant Kits (Tiangen, Beijing, China) from canola seeds at 2, 4, 6, and 8 WAP. RNA samples were quantified by NanoDrop 2000c Spectrophotometer (Thermo Scientific, Wilmington, DE, USA), and their integrity and purity confirmed using agarose gel electrophoresis. 1 μg of total RNA was reverse transcribed into cDNA using iScript cDNA Synthesis Kit (Bio-Rad, USA) according to the manufacturer’s protocol. PCR primers were designed with Primer 5.0 and Oligo 6.0 software. All RT-PCR reactions were performed using iTaq^TM^ Universal SYBR^®^ Green Supermix (Bio-Rad, USA) in a CFX96^TM^ Real-Time PCR Detection System. The qPCR cycling conditions were as follows: one cycle of 95°C for 30 s, then 39 cycles of 95°C for 5 s and 55–70°C for 1 min, followed by a melting curve ramping from 65 to 95°C with temperature increasing by 0.5°C every 5 s (one cycle). Data were analyzed using the 2^-ΔΔCT^ method (Livak and Schmittgen, 2001), normalized to beta-actin. Three replicates were used for each gene and data were analyzed using CFX Manager^TM^ v3.0.

## Results

### The Developmental Profiles of Oil Accumulation and Fatty Acids

**FIGURE 1:**
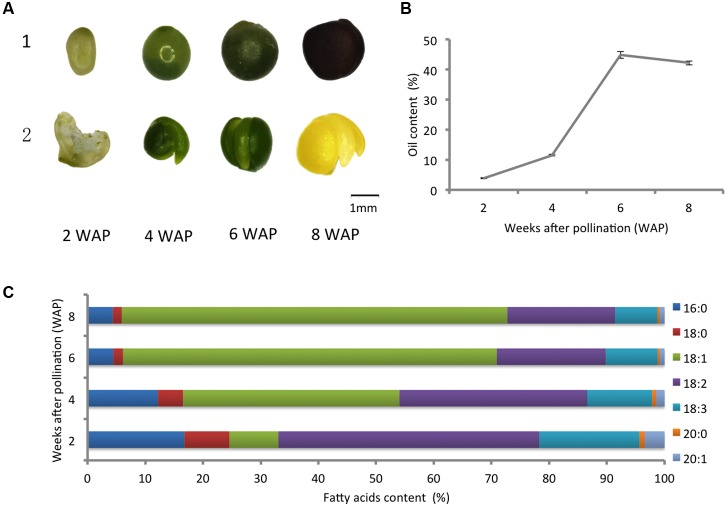
**Developmental profiles of oil accumulation and fatty acids. (A)** Morphological changes of canola: 1, seeds; 2, embryos (the first one is an opened seed since the embryo is too small). **(B)** The oil content of canola seeds at different points of development; the error bars represent the standard deviation of three replicates. **(C)** Changes in the content of fatty acids in developing canola seeds.

To obtain a comprehensive view of oil-related phenotypic changes during seed development in canola, we chose seeds at 2, 4, 6, and 8 WAP for the quantification of developmental profiles of oil accumulation and fatty acids. The pictures of seeds and embryos for these four developmental points are shown in **Figure 1A**. The content of oil and fatty acids of seeds were measured for four time points with three replicates at each point. The average oil content of three replicates is shown in **Figure 1B**. The values are reported as a weight percentage (weight of oil/weight of seed), and the error bars represent the standard deviation of three replicates. The oil content of seeds increased rapidly from 2 to 6 WAP, followed by a slight decrease at 8 WAP (**Figure 1B**). We measured the content of fatty acids in canola, including 16:0 (palmitic acid), 18:0 (stearic acid), 18:1 (oleic acid), 18:2 (linoleic acid), and 18:3 (linolenic acid), and characterized the developmental profiles of these fatty acids (**Figure 1C**). The values are expressed as a weight percentage of the total amount of fatty acids measured. A steady increase of the proportion of oleic acid (18:1) from 8.47 to 66.81% was observed, and it became the dominant form of fatty acid since 4 WAP (**Figure 1C**).

### RNA Sequencing, Alignment, and Quantification of Gene Expression

Total RNA from canola developing seeds at 2, 4, 6, and 8 WAP were isolated and sequenced separately by Illumina Hiseq 2000. After removing reads containing adapter, ploy-N and low quality reads from the raw sequencing data, approximately 1.97 billion paired-end 100-bp long high quality reads were generated. The four time points were represented by 58,826,944, 53,728,396, 42,234,736, and 42,297,630 reads, respectively. These high quality reads were deposited in the NCBI Sequence Read Archive (SRA^5^) database and available through Gene Expression Omnibus (GEO^6^) under accession GEO: GSE77637.

The sequence reads were aligned to the *B. napus* reference genome using TopHat Software, the statistics are shown in **Table 1**. Of the total reads from the four samples, 75.16, 76.45, 74.72, and 72.55% were mapped to a unique genomic location while 5.26, 4.64, 5.92, and 6.22% were matched to multiple locations, and the remaining 19.59, 18.91, 19.36, and 21.23% were unmatched. The gene expression levels of 101,040 genes, annotated with the publishing of the reference genome, were quantified and available at GEO under accession GEO: GSE77637.

**Table 1:** Alignment information of sequencing reads.

Sample	2 WAP	4 WAP	6 WAP	8 WAP
Total reads	58826944	53728396	42234736	42297630
Total mapped	47305246 (80.41%)	43568157 (81.09%)	34059288 (80.64%)	33319176 (78.77%)
Multiple mapped	3092829 (5.26%)	2490741 (4.64%)	2499862 (5.92%)	2630415 (6.22%)
Uniquely mapped	44212417 (75.16%)	41077416 (76.45%)	31559426 (74.72%)	30688761 (72.55%)
Read-1	22704425 (38.6%)	20740335 (38.6%)	16186396 (38.32%)	15725299 (37.18%)
Read-2	21507992 (36.56%)	20337081 (37.85%)	15373030 (36.4%)	14963462 (35.38%)
Reads map to +	22114905 (37.59%)	20515301 (38.18%)	15694409 (37.16%)	15375191 (36.35%)
Reads map to -	22097512 (37.56%)	20562115 (38.27%)	15865017 (37.56%)	15313570 (36.2%)
Non-splice reads	27022808 (45.94%)	25779653 (47.98%)	23155495 (54.83%)	21252302 (50.24%)
Splice reads	17189609 (29.22%)	15297763 (28.47%)	8403931 (19.9%)	9436459 (22.31%)


### Time-Series Differential Gene Expression Analysis

**FIGURE 2:**
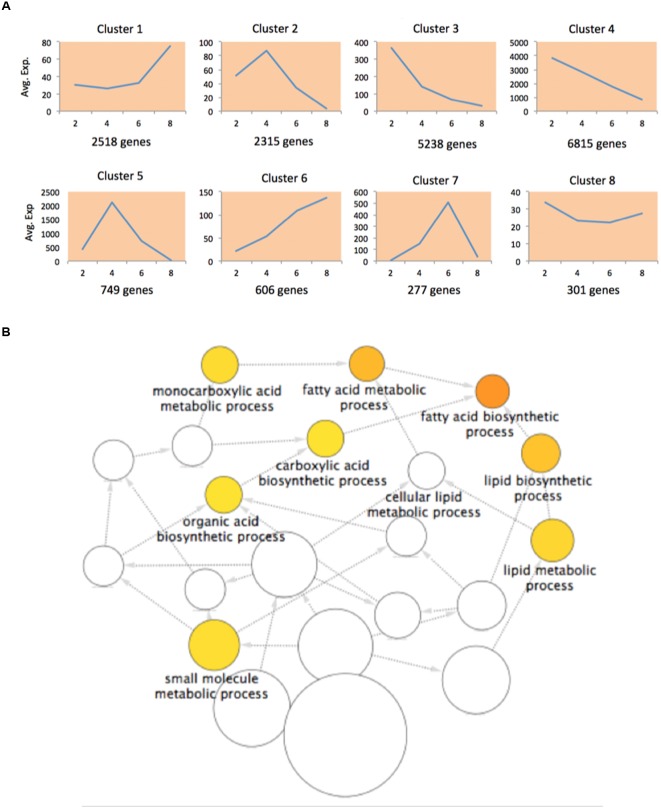
**Differentially expressed genes (DEGs) in transcriptomic analysis. (A)** The normalized average expression levels of DEGs in each cluster at 2, 4, 6, and 8 WAP. **(B)** Enriched GO terms in DEGs of cluster 5.

Since our data were collected at different time points, we performed a time-series differential expression analysis to investigate the global temporal patterns of transcriptomic changes, with a special focus on the dynamic changes in FA biosynthesis and degradation. We here define the differentially expressed genes (DEGs) across a time series as genes that are differentially expressed between any two time points. In total, we identified 18,819 DEGs listed in Supplementary Table S1. To gain biological understanding of these DEGs, we performed time-series expression profile clustering to search for common temporal expression patterns. We experimented with various numbers of clusters and found that eight clusters better captured the expression patterns of DEGs. Therefore, we classified all DEGs into eight co-expressed gene clusters using maSigPro (Nueda et al., 2014), all of these clusters and the corresponding gene members were listed in Supplementary Table S1. The genes in a cluster have similar temporal expression patterns and may involve in the same biological process. **Figure 2A** shows the average expression level of each cluster at each time point. We divided the time interval into three stages: 2-4, 4-6, and 6-8 WAP. Six broad classes become apparent across these stages: “up-up-up” (cluster 6), “up-up-down” (cluster 7), “up-down-down” (clusters 2, 5), “down-up-up” (cluster 1), “down-down-up” (cluster 8) and “down-down-down” (clusters 3, 4). Although, clusters 2 and 5 or clusters 3 and 4 belong to the same category, they show a clear difference (**Figure 2A**). To elucidate the enriched biological effects in these clusters, we carried out two types of enrichment analyses for each cluster: GO enrichment analysis and an enrichment analysis on pathways involved in acyl lipid metabolism (ALM).

**FIGURE 3:**
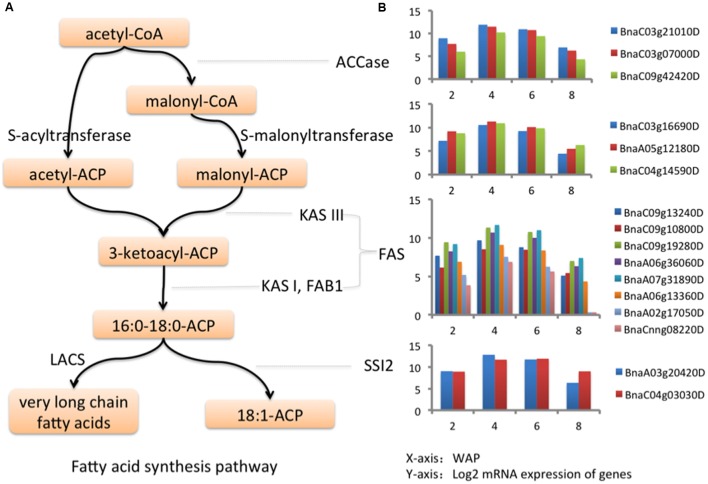
**Genes in fatty acid synthesis pathway. (A)** Fatty acid synthesis pathway. **(B)** The log2 expression values of key genes in fatty acid synthesis pathway at 2, 4, 6, and 8 WAP.

Gene ontology enrichment analysis is preferred for functional analysis of a list of genes. We performed GO enrichment analysis to identify significantly enriched GO terms in each cluster at the domain of “Biological Process” (BP). We identified 7, 22, 1, 52, and 8 significant GO biological processes at FDR < 0.0001 for clusters 1, 2, 3, 4, and 5, respectively, while none of processes were enriched in clusters 6-8. The results are shown in Supplementary Table S2. To avoid overly broad functional categories, we marked those GO terms higher than 1% within the genome in gray (Bargsten et al., 2014) and excluded them from further analyses (Supplementary Table S2). We found that the most significantly enriched GO biological process (FDR = 8.29E-09) in cluster 5 was fatty acid biosynthetic process (**Figure 2B**; Supplementary Table S2), which has a temporal expression pattern of “up-down-down” (**Figure 2A**). FA biosynthesis is the creation of fatty acids from acetyl coenzyme A (CoA), which is involved in several key steps, as shown in **Figure 3A**. Acetyl-CoA carboxylase (ACCase), catalyzing the formation of malonyl-CoA from acetyl-CoA, is considered as a rate-limiting enzyme in the first committed step in FA biosynthesis (Ohlrogge and Jaworski, 1997). In cluster 5, we identified 40 DEGs involved in FA biosynthesis, of which there are 3, 3, 8, and 2 genes corresponding to ACCase, *S*-acyltransferase, fatty acid synthase (FAS), and acyl-[acyl carrier protein] (ACP) desaturase (SSI2), respectively (Supplementary Table S3). FAS is a multi-enzyme system, including 3-ketoacyl-ACP synthase III (KAS III), 3-ketoacyl-ACP synthase I (KAS I), and 3-oxoacyl-ACP synthase II (KAS II or FAB1). The log2 expression values of these genes are shown in **Figure 3B**, all of them show an “up-down-down” expression pattern.

Cluster 2 has a similar pattern to cluster 5. The enriched GO processes in this cluster are mainly related to transport and cellular localization (Supplementary Table S2), which may represent the lipid transport and localization.

Cluster 6 has an expression pattern of “up-up-up” across three stages. Although, no significant GO terms were enriched in cluster 6, the genes encompassed may participate in the lipid storage. We found six annotated oleosins in 18,819 DE genes, three of which, BnaC09g27370D, BnaA05g21590D, and BnaA07g14970D, fell into this cluster. Moreover, the mRNA expression levels of these three oleosins were markedly up-regulated over time; their expression were increased approximately 14807, 502, 403-fold respectively at 8 WAP compared to 2 WAP.

Chalhoub et al. (2014) listed 1898 *B. napus* genes involved in ALM, and these genes were scattered in multiple pathways responsible for different facets of ALM. To examine the distribution of DEGs in these pathways, we calculated the enrichment of DEGs in each pathway, namely the number of DEGs falling into that pathway for each cluster.

**FIGURE 4:**
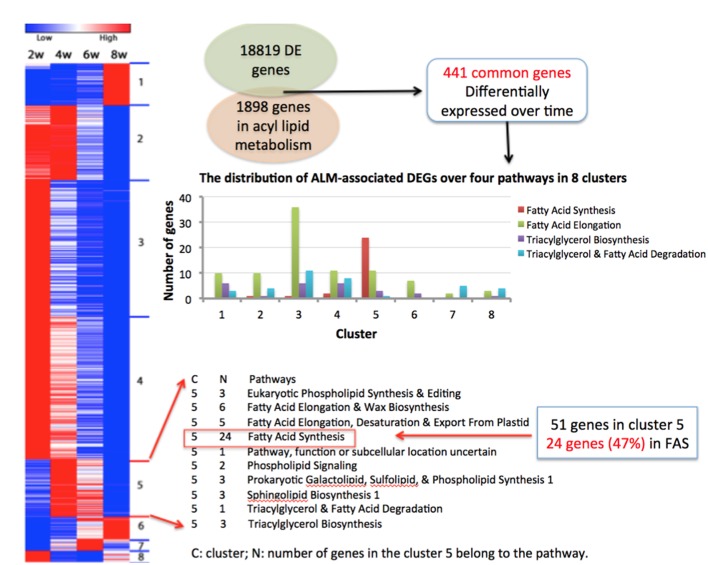
**Four hundred and forty one ALM-associated DEGs. (A)** The gene expression heat map of 441 ALM-associated DEGs and pathways in which the ALM-associated DEGs in cluster 5 were involved. **(B)** The distribution of ALM-associated DEGs over four pathways in eight clusters.

In total, there were 441 DEGs that were within the list of 1898 genes, and we designated these genes as ALM-associated DEGs (Supplementary Table S4). **Figure 4A** shows the gene expression heat map of 441 ALM-associated DEGs and pathways in which the ALM-associated DEGs in cluster 5 were involved. We found that ALM-associated DEGs in cluster 5 were enriched in the Fatty Acid Synthesis pathway. The cluster 5 contains 51 ALM-associated DEGs, of which 24 participate in FA synthesis and 11 are involved in FA elongation. In addition, there are a total of 28 ALM-associated DEGs that involve FA synthesis, of which 24 fall into the cluster 5 (**Figure 4B**), indicating that this cluster is responsible for the synthesis of fatty acids. These results are consistent with the observation in the GO enrichment analysis. ALM-associated DEGs relevant to Triacylglycerol and Fatty Acid Degradation were distributed within seven clusters (**Figure 4B**), implying that the FA degradation is a sophisticated process and regulated by different types of genes.

**FIGURE 5:**
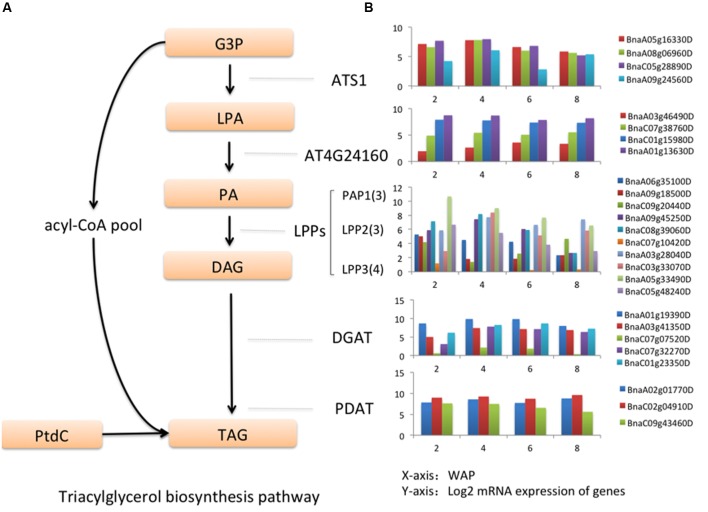
**Genes in triacylglycerol biosynthesis pathway. (A)** Triacylglycerol biosynthesis pathway. **(B)** The log2 expression values of key genes in triacylglycerol biosynthesis pathway at 2, 4, 6, and 8 WAP.

Triacylglycerol (TAG) is synthesized through three sequential acyl-CoA-dependent acylations of the glycerol backbone beginning with sn-glycerol-3-phosphate (G3P) (**Figure 5A**) (Lung and Weselake, 2006). The first two acylations, from G3P to lysophosphatidic acid (LPA) and from LPA to phosphatidic acid (PA), are catalyzed by glycerol-3-phosphate acyltransferase (ATS1) and lysophosphatidic acid acyltransferase (AT4G24160), respectively. Lipid phosphate phosphatases (LPPs) catalyze the dephosphorylation of PA to produce sn-1,2-diacylglycerol (DAG) prior to the final acylation catalyzed by diacylglycerol acyltransferase (DGAT). In addition, DAG can also be converted to TAG through the reaction catalyzed by phospholipid:diacylglycerol acyltransferase (PDAT) where the phosphatidylcholine (PtdC) is utilized as the acyl donor in TAG formation. We identified 26 canola genes involved in TAG biosynthesis (**Figure 5B**). Among them, almost all of ATS1 (BnaA05g16330D, BnaA08g06960D, BnaA09g24560D, BnaC05g28890D) and DGAT (BnaA01g19390D, BnaA03g41350D, BnaC01g23350D, BnaC07g07520D, BnaC07g32270D) have a temporal expression pattern of “up-down-down” (**Figure 5B**), which is consistent with the pattern we observed in FA biosynthesis pathway. The reaction catalyzed by DGAT has been considered as a committed step of TAG synthesis (Aznar-Moreno et al., 2015). Genes on the other nodes of the triacylglycerol biosynthesis pathway show multiple temporal patterns.

### Gene Set Association Analysis

The differential expression analyses of individual genes may fail to identify functional genes that are just moderately or slightly differentially expressed, while gene set association analysis can capture the effects of these genes since it measures the accumulative effect of multiple genes within a pathway. Gene set association analysis is powerful to reveal the biological pathways associated with a phenotype or condition. To identify pathways responsible for different stages of seed development, we conducted the gene set association analysis across the four time points: 2, 4, 6, and 8 WAP. We here used the gene expression level at 2 WAP as a reference status. We compared the gene expression profiles between 4-2, 6-2, and 8-2 WAP, and identified differentially expressed pathways associated with these stages.

The pathway annotation in *B. napus* is far from being completed while many biological pathways in *Arabidopsis thaliana* (*A. thaliana*) are well-characterized. Besides, most genes involved in lipid biosynthesis identified in the *A. thaliana* genome are conserved in *B. napus* (Chalhoub et al., 2014). Therefore, we mapped all of canola genes to their orthologous genes in *A. thaliana* for gene set association analysis. A total of 20,128 *A. thaliana* genes were matched with canola genes. We calculated a combined expression score for each *A. thaliana* gene by adding up the expression scores of all canola genes mapped into it (Supplementary Table S5).

The results of gene set association analyses are shown in **Table 2** and Supplementary Table S6. Significantly up-regulated KEGG pathways and GO biological processes were listed in **Table 2** for each of three comparisons: 4-2, 6-2, and 8-2 WAP. *p*-value, FDR, and family-wise error rate (FWER) were reported. We here used FDR < 0.05 as the cut-off value for assessing significance but also listed *p*-value and FWER for reference. The pathways in **Table 2** were sorted by FDR. In total, we identified 16 and 39 significantly up-regulated KEGG pathways and GO biological processes over the three stages, and the dynamic changes in these pathways were summarized as follows.

**Table 2:** Significantly up-regulated KEGG pathways and GO biological processes (FDR < 0.05).

Name	*p*-Value	FDR	FWER

**KEGG pathways**		
**4-2 WAP**		

G1	Fatty acid biosynthesis	0.000000	0.000000	0.000000
G2	Photosynthesis	0.000000	0.000000	0.000000
G3	Photosynthesis – antenna proteins	0.000000	0.000000	0.000000
G4	Biosynthesis of unsaturated fatty acids	0.000000	0.000409	0.001400
G5	Ubiquinone and other terpenoid-quinone biosynthesis	0.004880	0.026996	0.106800
G6	Valine leucine and isoleucine degradation	0.005210	0.041027	0.218000
G7	Porphyrin and chlorophyll metabolism	0.007639	0.046154	0.211400

**6-2 WAP**

G8	Biosynthesis of unsaturated fatty acids	0.000000	0.000000	0.000000
G9	Fatty acid biosynthesis	0.000000	0.000242	0.000600
G10	Stilbenoid diarylheptanoid and gingerol biosynthesis	0.000000	0.000248	0.000400
G11	Tropane piperidine and pyridine alkaloid biosynthesis	0.000370	0.001466	0.005000
G12	Limonene and pinene degradation	0.000365	0.013467	0.055600

**8-2 WAP**

G13	Limonene and pinene degradation	0.000000	0.000000	0.000000
G14	Stilbenoid diarylheptanoid and gingerol biosynthesis	0.000000	0.000000	0.000000
G15	Spliceosome	0.000000	0.001267	0.003800
G16	Peroxisome	0.000562	0.005445	0.020600

**GO biological processes**

**4-2 WAP**

G17	Photosynthesis	0.000000	0.000000	0.000000
G18	Lipid storage	0.000000	0.000000	0.000000
G19	Fatty acid biosynthetic process	0.000000	0.000078	0.000200
G20	Organ morphogenesis	0.000000	0.003289	0.011800
G21	Response to blue light	0.000000	0.010840	0.048000
G22	Embryonic development ending in seed dormancy	0.000000	0.016119	0.110600
G23	Chlorophyll biosynthetic process	0.001426	0.016237	0.097800
G24	Transmembrane receptor protein tyrosine kinase signaling pathway	0.000000	0.017746	0.091800
G25	Positive regulation of flower development	0.003541	0.030896	0.243800
G26	Response to cyclopentenone	0.003793	0.032157	0.274600
G27	Gravitropism	0.003247	0.033701	0.239800

**6-2 WAP**

G28	Lipid storage	0.000000	0.000130	0.000200
G29	Fatty acid biosynthetic process	0.000000	0.000260	0.000200
G30	Organ morphogenesis	0.000379	0.006517	0.017200
G31	Lignin biosynthetic process	0.001129	0.025337	0.083600
G32	Multicellular organismal development	0.000341	0.030200	0.123000
G33	Gravitropism	0.004120	0.044361	0.208600

**8-2 WAP**

G34	Response to heat	0.000000	0.000000	0.000000
G35	Response to high light intensity	0.000000	0.000000	0.000000
G36	Lipid storage	0.000000	0.000000	0.000000
G37	Response to hydrogen peroxide	0.000000	0.000108	0.000400
G38	Fatty acid beta-oxidation	0.000000	0.000135	0.000400
G39	Peroxisome organization	0.000939	0.004479	0.020000


#### The Dynamic Changes in the Biosynthesis of Fatty Acids and Unsaturated Fatty Acids

**FIGURE 6:**
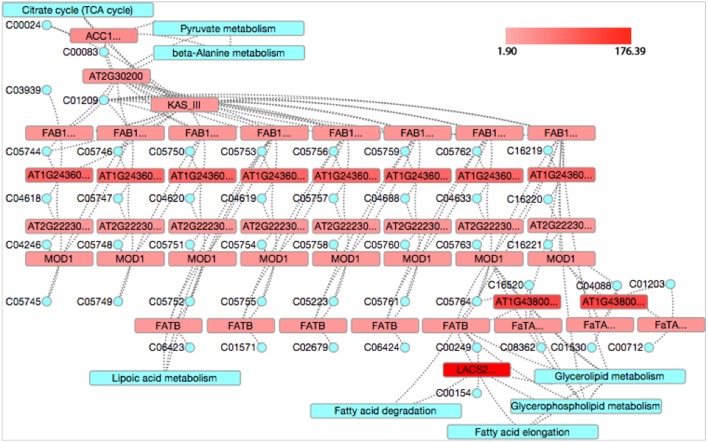
**The fold changes of genes in the fatty acid synthesis pathway.** The red color indicates up-regulation while green denotes down-regulation. The blue represents relevant other pathways. The tiny blue circle represents compounds that are a collection of small molecules, biopolymers, and other chemical substances that are relevant to biological systems.

Fatty acid synthesis was identified as a significant pathway at 4 and 6 WAP by both KEGG and GO analyses. It was ranked at top (FDR = 0) at 4 WAP and the second (FDR = 0.000242) at 6 WAP in the KEGG analysis, and it was at the third (FDR = 0.000078) at 4 WAP and the second (FDR = 0.00026) at 6 WAP in the GO analysis (**Table 2**), indicating FA synthesis is a dominant event from 2 to 6 WAP. We used the CytoKegg plug-in in the Cytoscape software (Shannon et al., 2003) to visualize the fold changes (FCs) of genes in the fatty acid synthesis pathway (**Figure 6**). The Fold change was calculated simply as the ratio of the gene expression value at the measured time point to the value at 2 WAP, and we set the fold changes of all genes at 2 WAP to 1. The fold change of a gene shown in **Figure 6** indicates the fold change value at its most perturbed time point. One KEGG node may correspond to multiple closely related genes that are subunits of a complex, and the symbols of these group nodes contain an ellipsis sign. For the group node including multiple genes, we took the maximum value of the fold changes of all genes as its fold change value. As shown in **Figure 6**, almost all genes in the fatty acid synthesis pathway were up-regulated, and the fold changes range from 1.9 to 176.39.

In canola seeds, the unsaturated fatty acids are the major forms of fatty acids. The proportion of unsaturated fatty acids is 91.84/6.08 from Sun’s study (Sun et al., 2011), and in our data it is approximately 94% at 8 WAP. Similar to the fatty acid synthesis pathway, the unsaturated fatty acid synthesis pathway was also identified as significance at 4 and 6 WAP by KEGG analysis (**Table 2**). It was ranked at fourth (FDR = 0.000409) at 4 WAP and the top (FDR = 0) at 6 WAP, indicating the synthesis of unsaturated fatty acids is a dominant event in seed development from 2 to 6 WAP as well. In addition, our results show that fatty acid synthesis is the most dominant process at stage 1 while unsaturated fatty acid synthesis is the most active process at stage 2. This is consistent with the order of molecular events in seed development.

#### The Dynamic Changes in the Degradation of Fatty Acids and Unsaturated Fatty Acids

Although genes for the biosynthesis of saturated and unsaturated fatty acids were up-regulated significantly by 6 WAP compared to the initial level at 2 WAP, they rapidly fell into a low level at the last stage of 6 to 8 WAP. Conversely, it seems that the degradation of fatty acids governs the last stage. One KEGG pathways, PEROXISOME (FDR = 0.005445), and two GO processes, FATTY ACID BETA-OXIDATION (FDR = 0.000135) and PEROXISOME ORGANIZATION (FDR = 0.004479), were related to the degradation of fatty acids and unsaturated fatty acids. The degradation of fatty acids and unsaturated fatty acids in most organisms occurs primarily via the β-oxidation cycle. In plants, β-oxidation is the catabolic process by which fatty acid molecules are broken down in the peroxisomes to generate acetyl-CoA, which enters the citric acid cycle (Poirier et al., 2006).

#### The Dynamic Changes in Other Pathways Related to Fatty Acid Synthesis

Except for the pathways directly involved in the biosynthesis of saturated and unsaturated fatty acids, there are several other pathways that may be relevant to FA synthesis indirectly. The pathway related to lipid storage (LIPID STORAGE) was significantly up-regulated across all stages, possibly responding to the production of large amounts of oil. Two pathways representing photosynthesis, PHOTOSYNTHESIS and PHOTOSYNTHESIS-ANTENNA PROTEINS, were substantially up-regulated in the 2-4 WAP stage. Besides, there were another two light-related pathways (RESPONSE TO BLUE LIGHT, RESPONSE TO HIGH LIGHT INTENSITY) that were up-regulated in the 4-6 and 6-8 WAP stages, respectively. Fatty acid synthesis is closely related to photosynthesis. The synthesis of malonyl-CoA, the first committed step in FA synthesis, is regulated by light (Sasaki et al., 1997).

### Time-Series Proteomic Analysis

**FIGURE 7:**
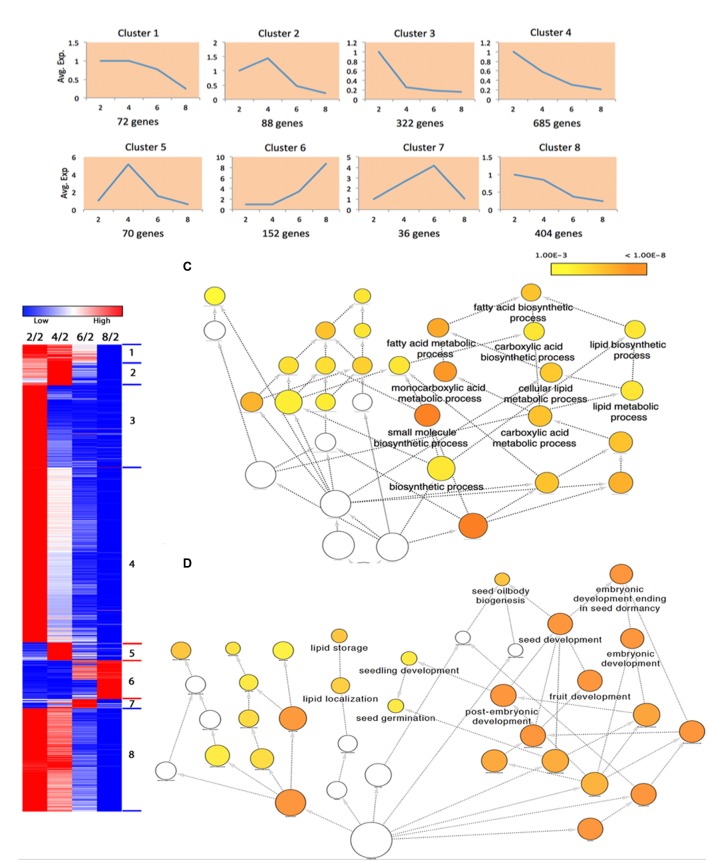
**Differentially expressed proteins (DEPs) in proteomic analysis. (A)** The average relative expression levels of DEPs in each cluster at 2, 4, 6, 8 WAP. **(B)** The expression heat map of DEPs. **(C)** Enriched GO terms in DEPs of cluster 5. **(D)** Enriched GO terms in DEPs of cluster 6.

The whole proteome of canola seeds were measured at the same four time points and for same seed samples as the transcriptome. In total, we detected 2651 proteins, and their relative expression levels are shown in Supplementary Table S7. By visual inspection, we identified 78 proteins that involved lipid metabolism. Among 2651 proteins, 1829 were identified as differentially expressed proteins (DEPs) (Supplementary Table S8). We here define a DEP as a protein whose expression level is at least 3-fold increased or decreased compared with that at 2 WAP at any time point. These DEPs were grouped into eight co-expressed clusters (Supplementary Table S8). The temporal change of the average relative expression level of each cluster at each time point is shown in **Figure 7A**, and the expression heat map of these DEPs is shown in **Figure 7B**. Our results show that there exist five patterns across the three stages: “up-up-up” (cluster 6), “up-up-down” (cluster 7), “up-down-down” (clusters 2, 5), “down-down-down” (clusters 3, 4, 8), and “flat-down-down” (cluster 1). As expected, we found that the average expression levels of genes and the average relative expression levels of the proteins are highly positively correlated for clusters 2-7; the Pearson correlation coefficients are 0.98, 0.98, 0.97, 0.99, 0.90, and 0.97, respectively, implying these clusters may involve similar cellular processes. It shows a negative correlation for the cluster 1, and the correlation coefficient is -0.97.

We performed the GO enrichment analysis to uncover the enriched biological effects in these protein clusters; the results are shown in Supplementary Table S9. We found that the fatty acid biosynthetic process (FDR = 4.46E-06) and several related terms enriched with DEPs in cluster 5 (**Figure 7C**); this is consistent with the effects that enriched with DEGs in cluster 5. We found nine DEPs that involved the FA biosynthesis in this cluster (Supplementary Table S8), including two ACCase (224814586, 14388188) and three acyl-ACP desaturase (674888444, 674906651, 544370319). ACCase is a key enzyme that regulates the rate of the FA biosynthesis (Ohlrogge and Jaworski, 1997), while acyl-ACP desaturase is responsible for the conversion of saturated fatty acids to unsaturated fatty acids (Zhang et al., 2015).

Seed oilbody biogenesis (FDR = 1.63E-06), lipid storage (FDR = 2.05E-06), and lipid localization (FDR = 6.05E-06) are three significantly enriched terms in cluster 6 (**Figure 7D**). In the proteomic analysis, we identified a total of 12 oleosins that were differentially expressed, and all of them are located in the cluster 6 (Supplementary Table S8), indicating proteins in this cluster may play important roles in the seed oilbody biogenesis.

All 2051 proteins have corresponding gene expression levels. We identified 41 genes that were differentially expressed in both transcriptomic analysis and proteomic analysis (Supplementary Table S10). Among them, BnaC03g21010D (ACCase), BnaA09g03610D (ACP), BnaA03g20420D (acyl-ACP desaturase), BnaC09g27370D (oleosin), and BnaC04g45790D (non-specific lipid-transfer protein) were related to lipid metabolism.

### Quantitative Real-Time PCR (qRT-PCR) Analysis

**FIGURE 8:**
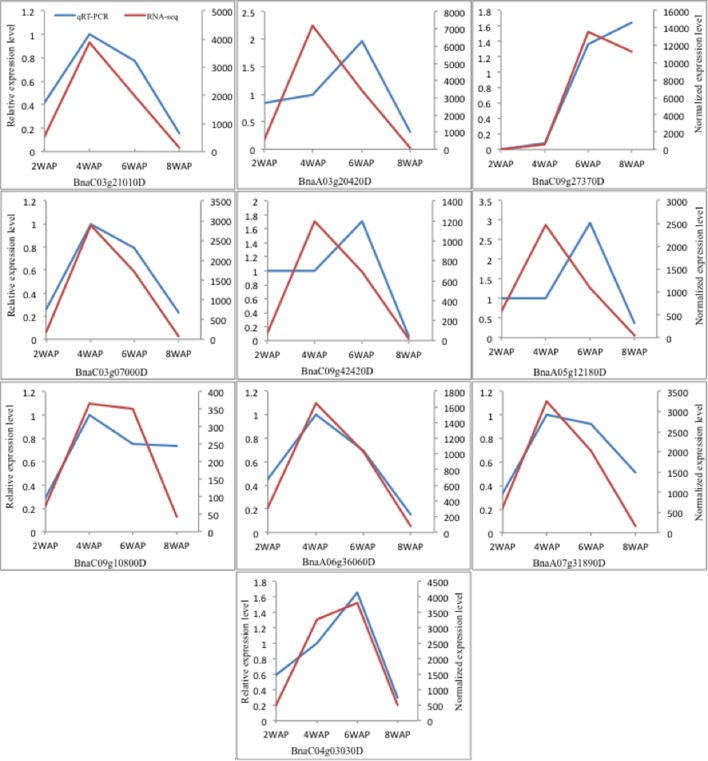
**The expression levels of 10 genes at 2, 4, 6, and 8 WAP for qRT-PCR and RNA-Seq experiment**.

To confirm the temporal expression patterns revealed by the transcriptomic analyses, 10 genes involved in FA biosynthesis were chosen for qRT-PCR analyses, including three ACCases (BnaC03g21010D, BnaC03g07000D, BnaC09g42420D), *S*-malonyltransferase (BnaA05g12180D), KAS III (BnaC09g10800D), KAS I (BnaA06g36060D), KAS II (BnaA07g31890D), two SSI2 (BnaA03g20420D, BnaC04g03030D), and oleosin (BnaC09g27370D). Three of them, BnaC03g21010D, BnaA03g20420D, and BnaC09g27370D, were detected as differentially expressed by both transcriptomic analysis and proteomic analysis (Supplementary Table S10). All others were identified as DEGs by transcriptomic analysis. Except for BnaC04g03030D and BnaC09g27370D, which have an “up-up-down” pattern, all remaining genes show “up-down-down” pattern in the RNA-Seq experiment. qRT-PCR results are shown in **Figure 8**. We found that the temporal expression patterns of six genes, BnaC03g21010D, BnaC03g07000D, BnaC09g10800D, BnaA06g36060D, BnaA07g31890D, and BnaC04g03030D, were confirmed by qRT-PCR analysis. We observed a shift between peaks of two expression profiles for three genes: BnaA03g20420D, BnaC09g42420D, and BnaA05g12180D. The patterns of BnaC09g27370D are same for the first two stages while they are different at the third stage. So, in most cases, qRT-PCR results were consistent with the observations from the transcriptomic experiments.

## Discussion

Unraveling the mechanism underlying the lipid metabolism is vital for genetic engineering of canola to increase oil yield and/or modify oil composition. Lipid biosynthesis consists of two phases: FA synthesis and TAG assembly. In this study, we conducted a joint analysis of transcriptome and proteome to uncover the dynamic changes in FA biosynthesis and degradation during seed development. The temporal changes of gene expression and protein profiles were measured across three stages, 2-4, 4-6, and 6-8 WAP. Our results show that the FA biosynthesis is a dominant cellular process during the first two stages, while the degradation mainly happens in stage 3. We found that the two nodes, ACCase and acyl-ACP desaturase, might be critical for the FA biosynthesis pathway in canola since significant changes on these nodes were detected by both transcriptomic and proteomic analyses. ACCase catalyzes the irreversible reaction from acetyl-CoA to malonyl-CoA, which is vital for the initiation of FA biosynthesis and has been widely accepted as a key enzyme controlling the rate of FA biosynthesis (Ohlrogge and Jaworski, 1997). Acyl-ACP desaturase is a core enzyme that catalyzes the conversion of saturated fatty acids to unsaturated fatty acids (Zhang et al., 2015). It has been found that the canola oil has over 90% unsaturated fatty acids (Sun et al., 2011), and from our data it is approximately 94% at 8 WAP. This might be the reason that acyl-ACP desaturases were extraordinarily up-regulated during the period of FA synthesis.

We identified many well-known genes that involved lipid metabolism such as ACCase. In addition, our results also revealed many potential novel candidates that might play important roles in lipid metabolism. We found that the GO term, fatty acid biosynthetic process, was enriched in cluster 5. There are 749 DEGs in this cluster (Supplementary Table S1). Except for known genes, some of remaining genes might also contribute to lipid biosynthesis since genes in a cluster have similar temporal change patterns and some may be functional in the same cellular process. Our study provides a starting point for further investigating the potential roles of these genes in fatty acid biosynthesis.

Xu et al. (2015) compared the gene expression profiles of *B. napus* pods at 5-7 (1), 15-17 (2), and 25-27 (3) days after flowering (DAF). In general, we expect to see the up-regulation of genes involved in FA biosynthesis since oil is accumulated rapidly during the development of pod. According to Table 4 in Xu’s paper which listed the differentially expressed oil-related homologous genes across three stages for the two varieties (Sollux and Gaoyou), they detected a total of 21 up-regulated lipid-related DEGs. Among these DEGs, many are related to phospholipid signaling pathway, and only one, MCMT (Malonyl-CoA), is directly involved in FA biosynthesis. However, from our analysis, we see the up-regulation of genes on almost every node of FA biosynthesis pathway during 2 to 4 WAP. This inconsistency may be caused by the different constitutions between pod and seed. The transcriptomes of seeds might be more suited for characterizing the molecular mechanisms responsible for oil content and composition since the gene expression is region-specific and subregion-specific (Belmonte et al., 2013; Borisjuk et al., 2013). Even seeds are highly organized structures that consist of embryo, endosperm, and seed-coat regions, and each region can be further divided into morphologically distinct subregions. Belmonte et al. (2013) analyzed the gene expression profiles of regions and subregions of *Arabidopsis* seeds throughout development, and they found that there were functional differences between subregions and genes expressed specifically within a subregion appeared to play a significant role in specifying its function. In addition, another study (Borisjuk et al., 2013) demonstrated that the metabolic fluxes in embryo, the *B. napus* oil-producing tissue, were regulated by seed architecture for the efficient use of available light and space. So, investigating the profiles of gene activity of different regions and the coordinated development of these regions may enable an integrated understanding of the processes underlying seed development in canola, and we plan to explore this domain in the near future.

Very few RNA-Seq studies have been reported concerning the seed oil regulation in canola. A study (Deng et al., 2015) analyzed the transcriptional profiles of developing canola embryos collected at 17, 29, 35, 43, and 52 days of pollination (DAP) using RNA-Seq method, and characterized the temporal expression patterns of DEGs involved in carbon flow to acetyl-CoA, acetyl-CoA to fatty acids, and phytohormone related DEGs. The expression patterns of DEGs involved in fatty acid biosynthesis in this investigation were similar to those patterns observed by our study. Although, both studies carried out GO and KEGG analyses, the way we did it was different. Deng et al. (2015) simply assigned DEGs to GO and KEGG pathways while we assessed the associations of all 83 KEGG pathways and 201 GO biological processes with the different stages of seed development and identified the most significant pathways. Our method could more accurately and comprehensively identify the biological processes significantly changed during seed development.

It was assumed, based on the central dogma, that there is a direct positive correlation between mRNA and protein abundances. However, many studies have shown that the correlation can be low due to various factors (de Sousa Abreu et al., 2009; Ghazalpour et al., 2011; Vogel and Marcotte, 2012). Although, we demonstrated that there were significant correlations between transcriptomic and proteomic changes at the cluster/gene set level and with regard to enriched GO terms, a relatively small overlap was observed in terms of DEGs. This is possibly because of (1) post-transcriptional and translational regulation or protein degradation; (2) a limited number of proteins detected; (3) difficulty to detect, identify and quantify low-abundance proteins; (4) a delay between mRNA and protein accumulation (Gedeon and Bokes, 2012). The potential discrepancy between the abundances of mRNA and the corresponding protein favors an integrative approach to comprehensively decipher the regulation of cellular processes at both transcriptional and proteomic dimensions. Moreover, our integrative analysis also provides insights into the regulatory programs that may not be uncovered from individual analyses of mRNA or protein expressions; for example, we detected more oleosins at the protein level, and it reveals a group of genes involved the oilbody biogenesis.

Our seed samples were harvested at 2, 4, 6, 8 WAP, and this time interval should be a good capture for the dynamic change in FA biosynthesis. Hajduch et al. (2006) have conducted a study to analyze the *B. napus* seed proteins at 2, 3, 4, 5, 6 weeks after flowering (WAF). They reported a pattern of “up-up-down-down” across the four stages, very similar to what we observed in our transcriptional and proteomic analyses, with regard to the temporal changes of genes involved lipid biosynthesis. The mRNA and proteins of these genes increased rapidly from 2 until 4 WAF followed by a decrease at 6 WAF, indicating 2-6 WAF or WAP is suited for characterizing the dynamic process relative to FA biosynthesis. However, 2-8 WAP may be not long enough for monitoring the entire process of degradation. We found that the expression profiles of genes participating in FA degradation show several types of distinct patterns, and some of them have a pattern of “up-up-up,” implying that fatty acids may be still undergoing degradation after 8 WAP, so it may take longer to measure the change in FA degradation.

We compared the temporal expression profiles of 10 genes from qRT-PCR and RNA-Seq and observed a shift between peaks of two expression profiles for three genes: BnaA03g20420D, BnaC09g42420D, and BnaA05g12180D. This difference may be caused by the alternative splicing during pre-mRNA processing process. qRT-PCR only measures the expression level of a segment of a gene while RNA-Seq can more comprehensively capture the expression level of the whole gene. Alternative splicing might cause the different expression of different regions of a gene, resulting in the difference between two expression profiles.

Except for the time-series differential expression analysis of individual genes, we also employed a gene set association analysis to measure the genome-wide changes at the pathway level. This approach can not only offer a systems-level view of gene networks regulating dynamic cellular processes, but also capture the subtle changes of multiple genes with moderate or weak effects, which could not be detected by individual gene analysis due to the tremendous amount of noise in genomic data. For example, in our gene set association analysis, the results show that “fatty acid biosynthesis” is the most significant pathway at stage 1 (2-4 WAP) while “biosynthesis of unsaturated fatty acids” is on the top at stage 2 (4-6 WAP) (**Table 2**); this is in agreement with the order of molecular events. The fatty acids are substrates for the biosynthesis of unsaturated fatty acids, so there should be a delay between the peaks of mRNA levels of fatty acid related genes and unsaturated fatty acid related genes.

In this study, we used *A. thaliana* pathways for gene set analysis. This is reasonable since studies have shown that there exists a common mechanism underlying lipid metabolism regulation between *B. napus* and *A. thaliana* (Niu et al., 2009). Moreover, most genes involved in lipid biosynthesis identified in the *A. thaliana* genome are conserved in *B. napus* (Chalhoub et al., 2014).

## Author Contributions

WQ supervised the project; QX and WQ designed the methods; HW, YC, HD, CZ, and QX performed experiments; HW, YC, YD, JM, WZ, SW, YL, JL, and QX analyzed data; HW and QX wrote the manuscript; All authors read and approved the final manuscript.

## Conflict of Interest Statement

The authors declare that the research was conducted in the absence of any commercial or financial relationships that could be construed as a potential conflict of interest.

## Footnotes

1 https://apps.fas.usda.gov/psdonline/circulars/oilseeds.pdf

2 http://www.genoscope.cns.fr/brassicanapus/data

3 http://ccb.jhu.edu/software/tophat/index.shtml

4 http://www-huber.embl.de/users/anders/HTSeq/doc/count.html

5 http://www.ncbi.nlm.nih.gov/sra

6 http://www.ncbi.nlm.nih.gov/geo
